# Supplementary material for: ErbB signaling is a potential therapeutic target for vascular lesions with fibrous component
Source: eLife. 2023 May 18;12:e82543. doi: 10.7554/eLife.82543 (PMC10260011; doi:10.7554/eLife.82543)
Supplement: Figure 2—figure supplement 1—source data 1. [file elife-82543-fig2-figsupp1-data1.zip › Fig 2 - figure supplement 1 - source file/Fig 2 - figure supplement 1 - source file.pdf]

# Full unedited gels for Fig 3 – figure supplement 1D

**CD31**

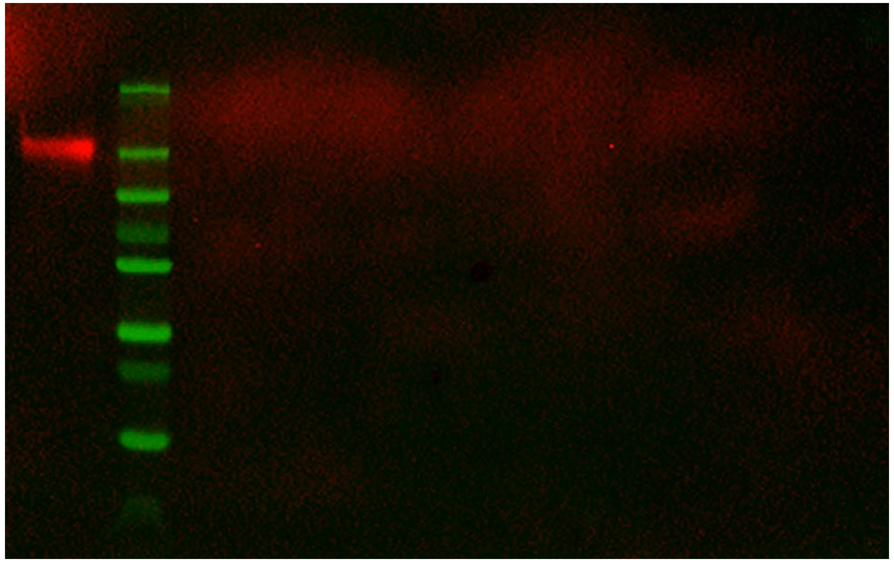

**β-actin**

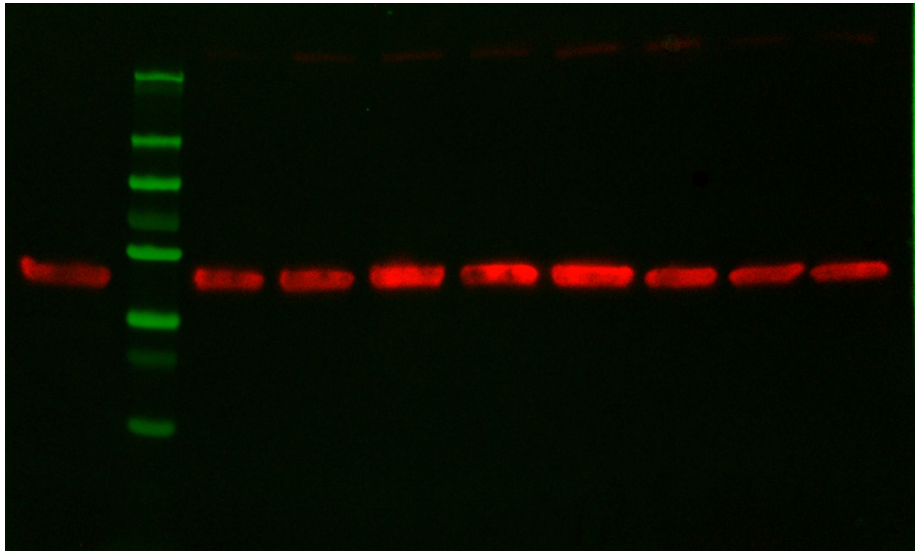

2nd sample from right excluded due to pathological diagnosis --> Atypical VM; some features of Maffucci syndrome

These lanes were included in the Figure

These lanes were included in the Figure

# Full unedited gels for Fig 3 – figure supplement 1E

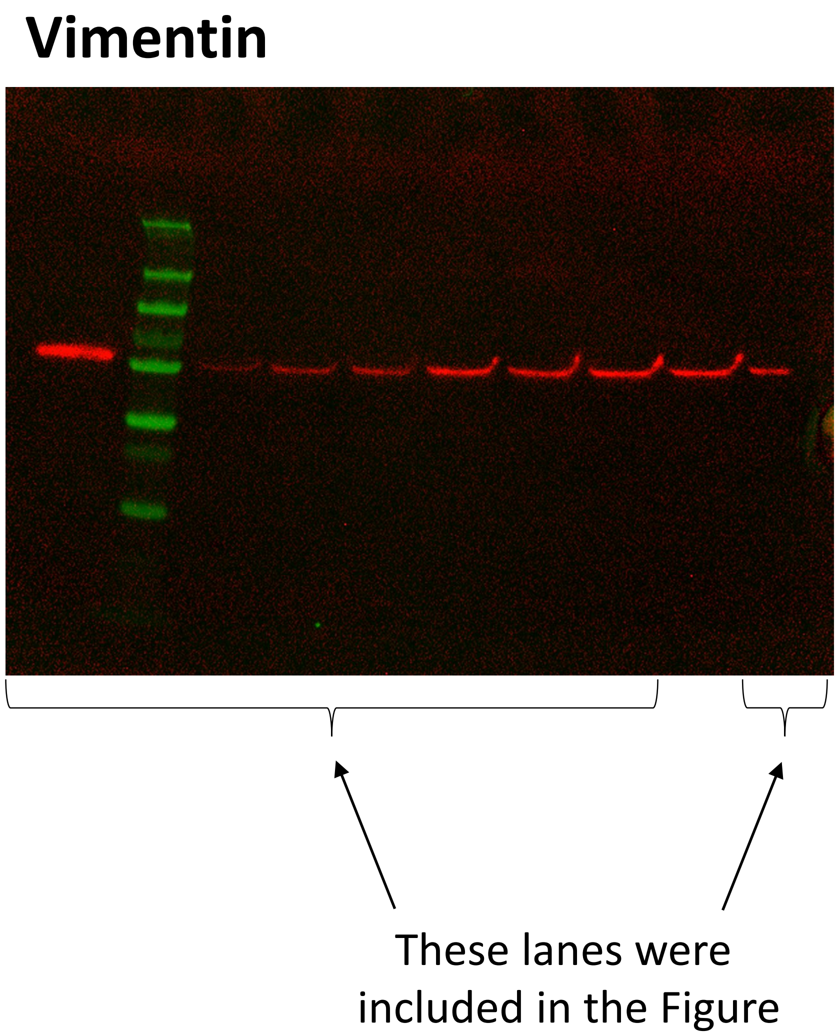


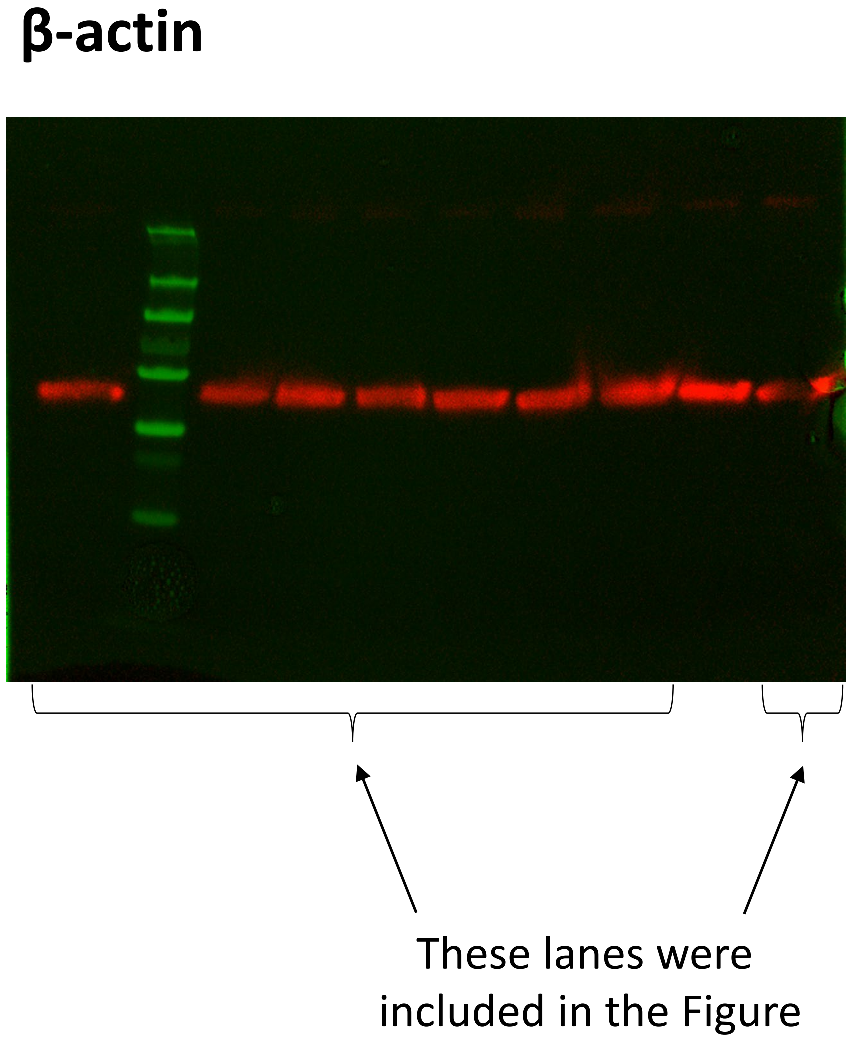


2nd sample from right excluded due to pathological diagnosis --> Atypical VM; some features of Maffucci syndrome

# Full unedited gels for Fig 3 – figure supplement 1F

**αSMA**

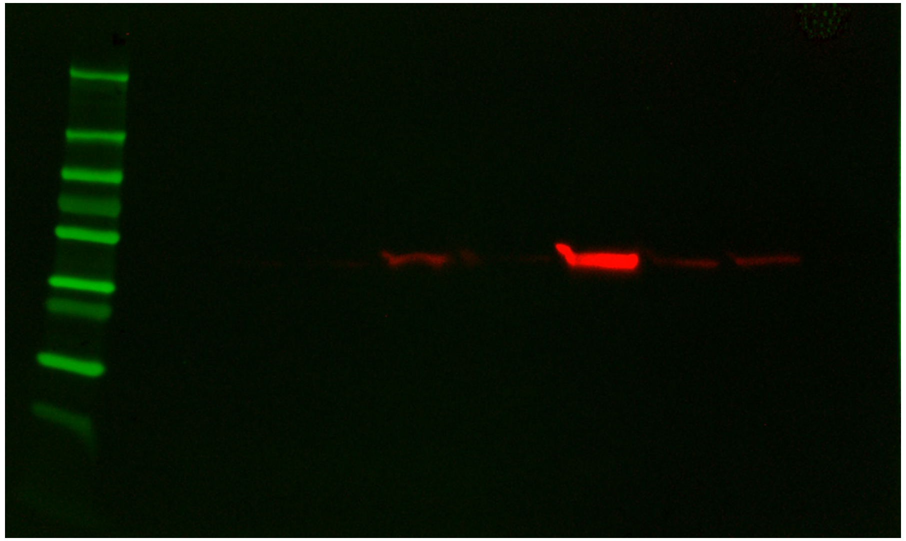

**β-actin**

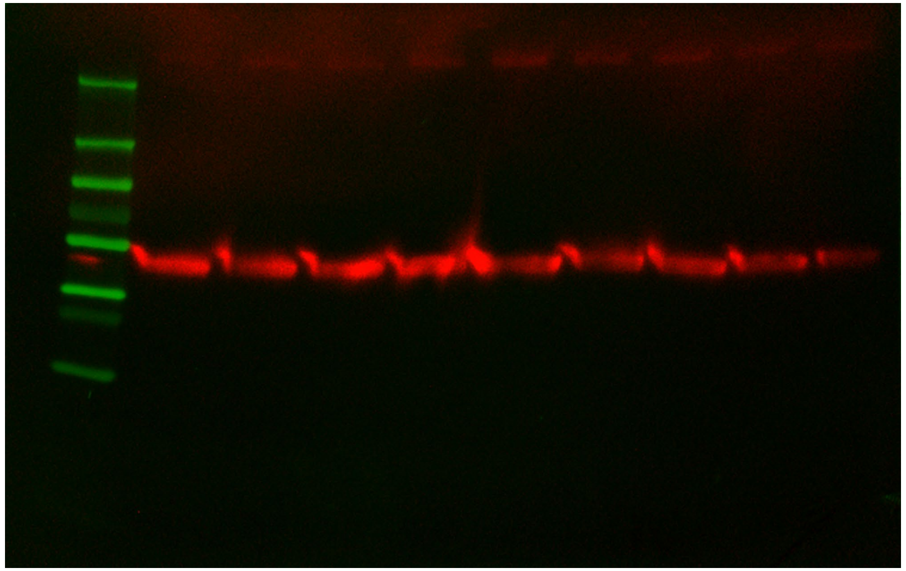

2nd sample from right excluded due to pathological diagnosis --> Atypical VM; some features of Maffucci syndrome

These lanes were included in the Figure

These lanes were included in the Figure
