# Supplementary figures and images for: ErbB signaling is a potential therapeutic target for vascular lesions with fibrous component
Source: eLife. 2023 May 18;12:e82543. doi: 10.7554/eLife.82543 (PMC10260011; doi:10.7554/eLife.82543)

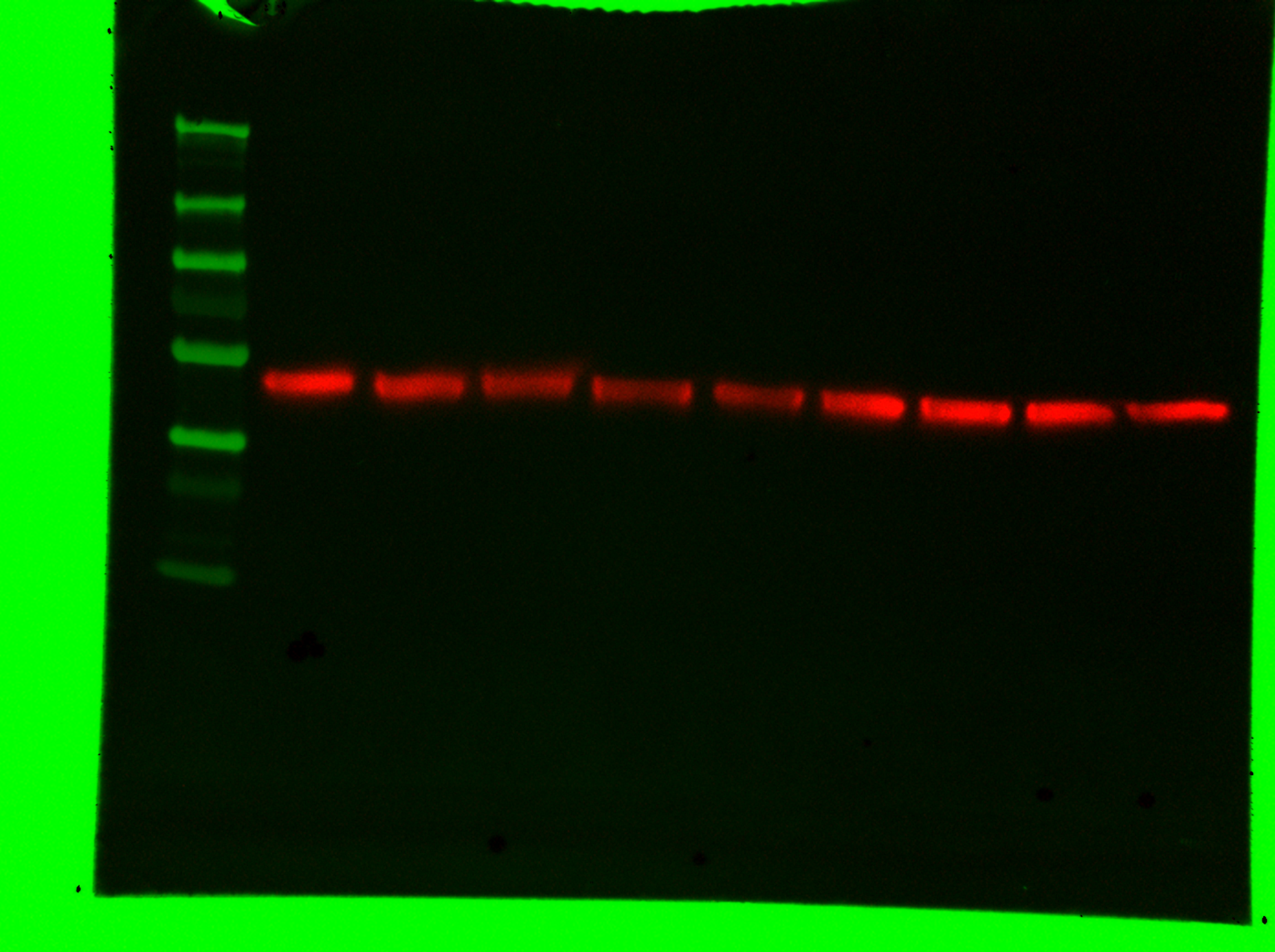

Supplement: Figure 2—figure supplement 1—source data 1. [file elife-82543-fig2-figsupp1-data1.zip › Fig 2 - figure supplement 1 - source file/Fig 2 - figure supplement 1E - source file_b-actin.tif]

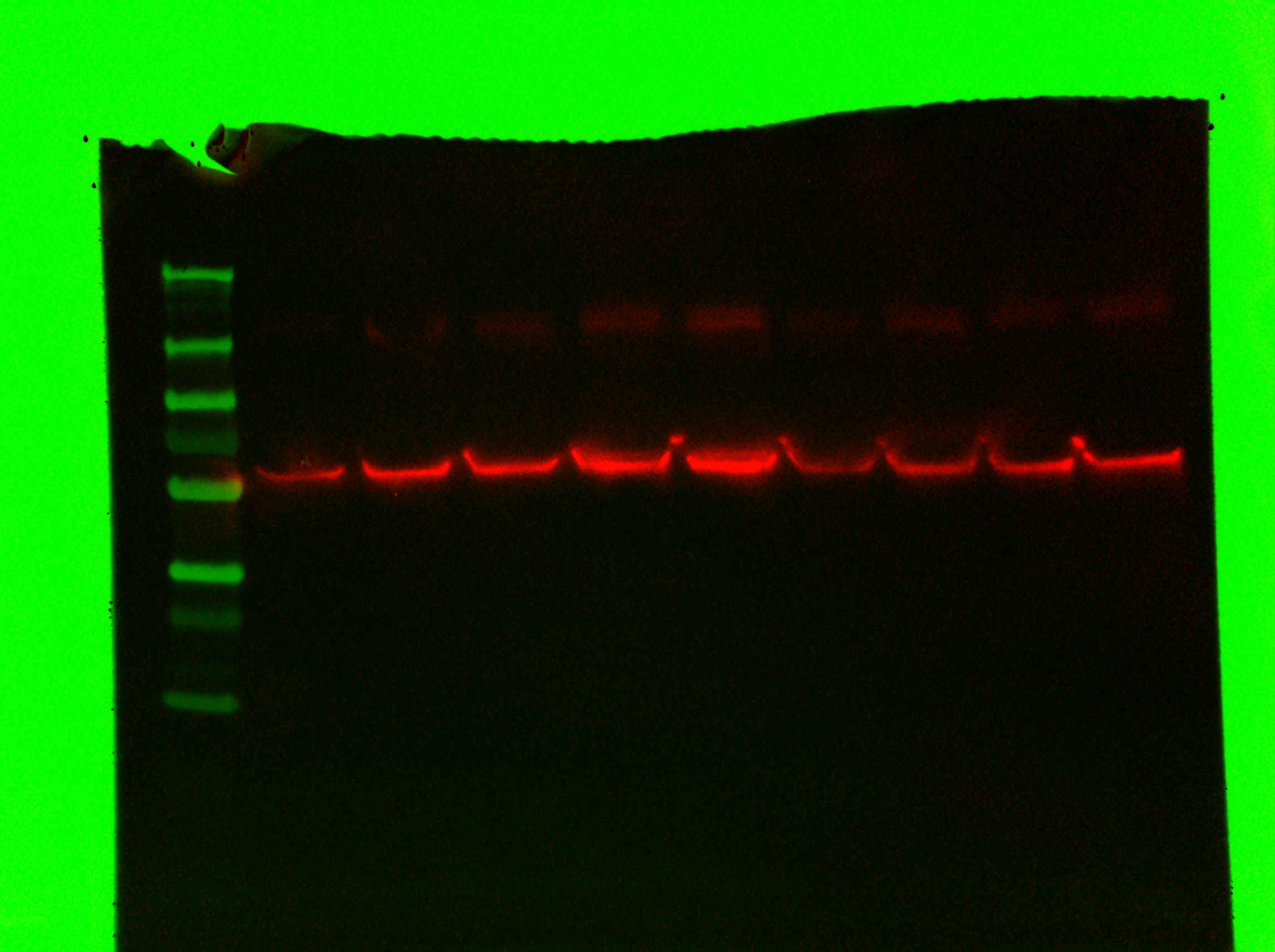

Supplement: Figure 2—figure supplement 1—source data 1. [file elife-82543-fig2-figsupp1-data1.zip › Fig 2 - figure supplement 1 - source file/Fig 2 - figure supplement 1E - source file_vimentin.tif]

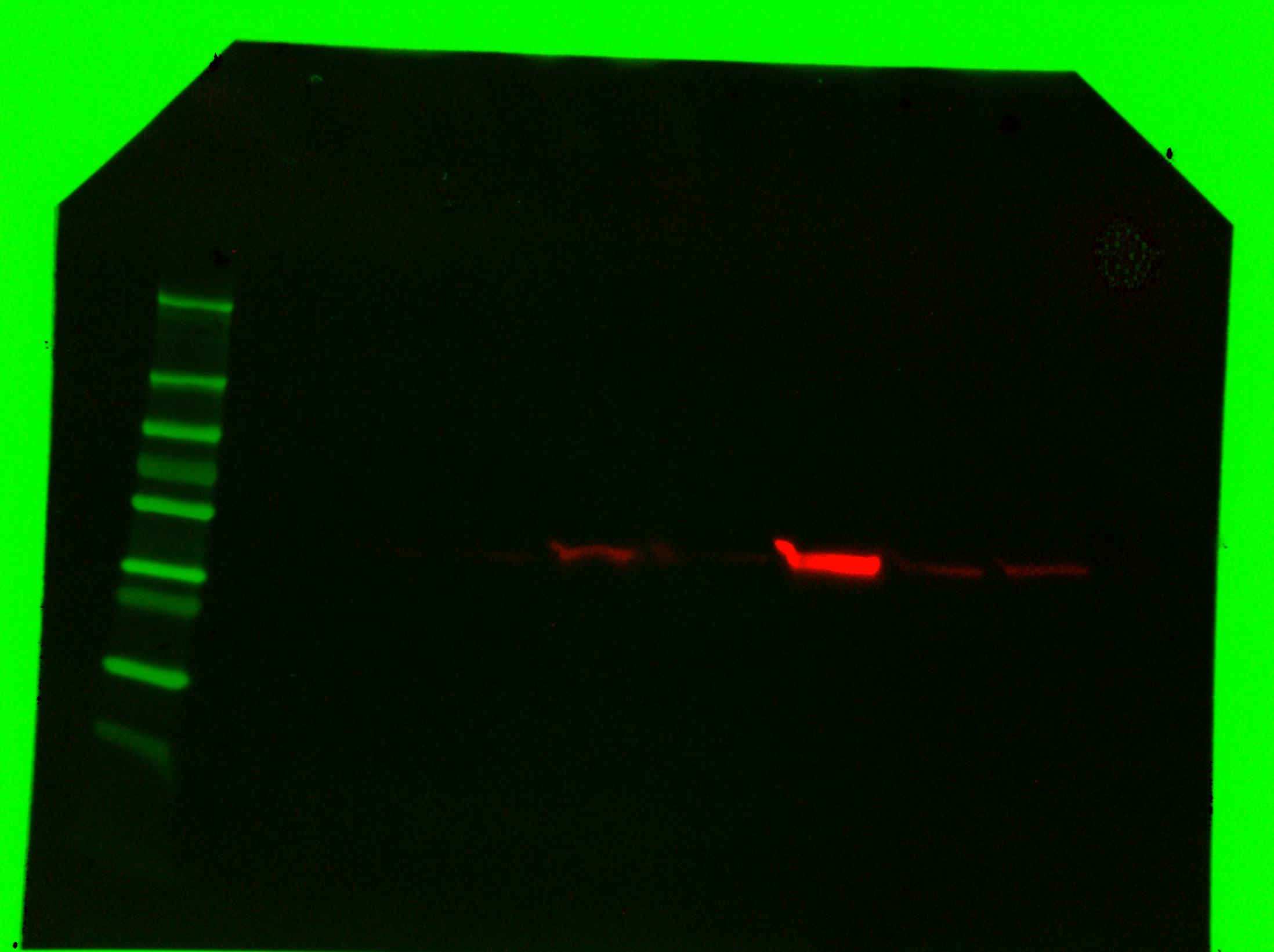

Supplement: Figure 2—figure supplement 1—source data 1. [file elife-82543-fig2-figsupp1-data1.zip › Fig 2 - figure supplement 1 - source file/Fig 2 - figure supplement 1F - source file_aSMA.tif]

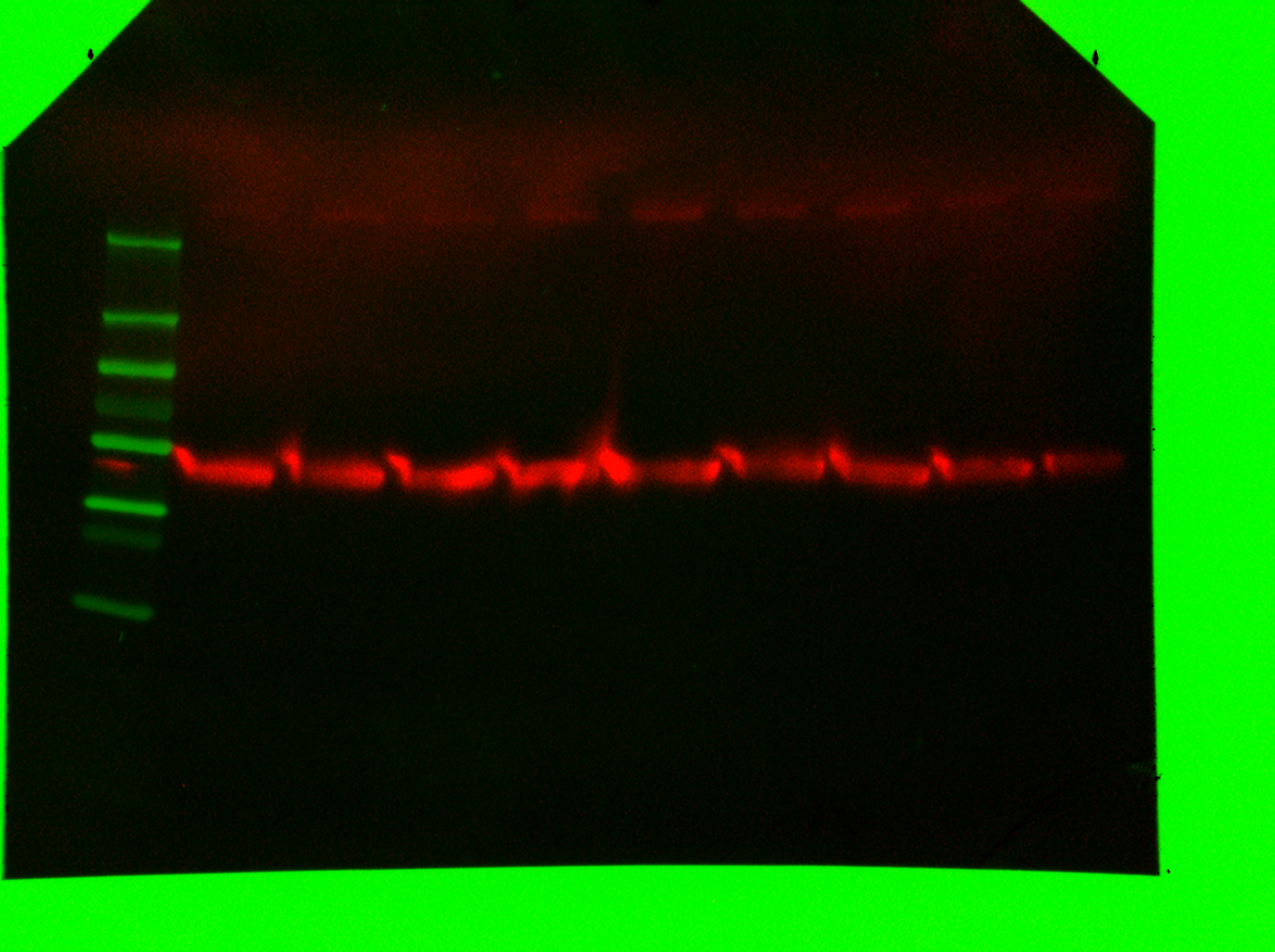

Supplement: Figure 2—figure supplement 1—source data 1. [file elife-82543-fig2-figsupp1-data1.zip › Fig 2 - figure supplement 1 - source file/Fig 2 - figure supplement 1F - source file_b-actin.tif]

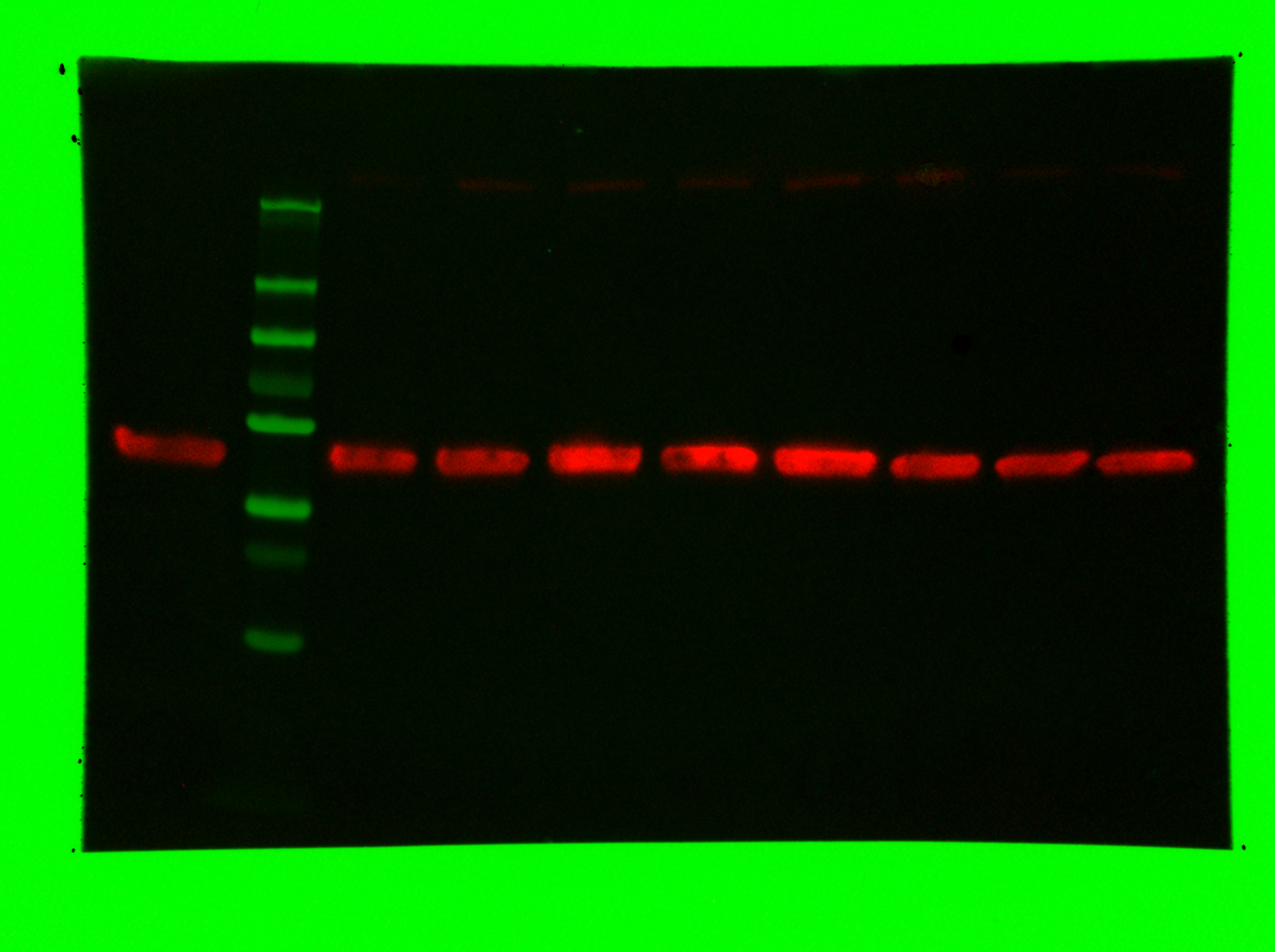

Supplement: Figure 2—figure supplement 1—source data 1. [file elife-82543-fig2-figsupp1-data1.zip › Fig 2 - figure supplement 1 - source file/Fig2 -figure supplement 1D - source file_b-actin.tif]

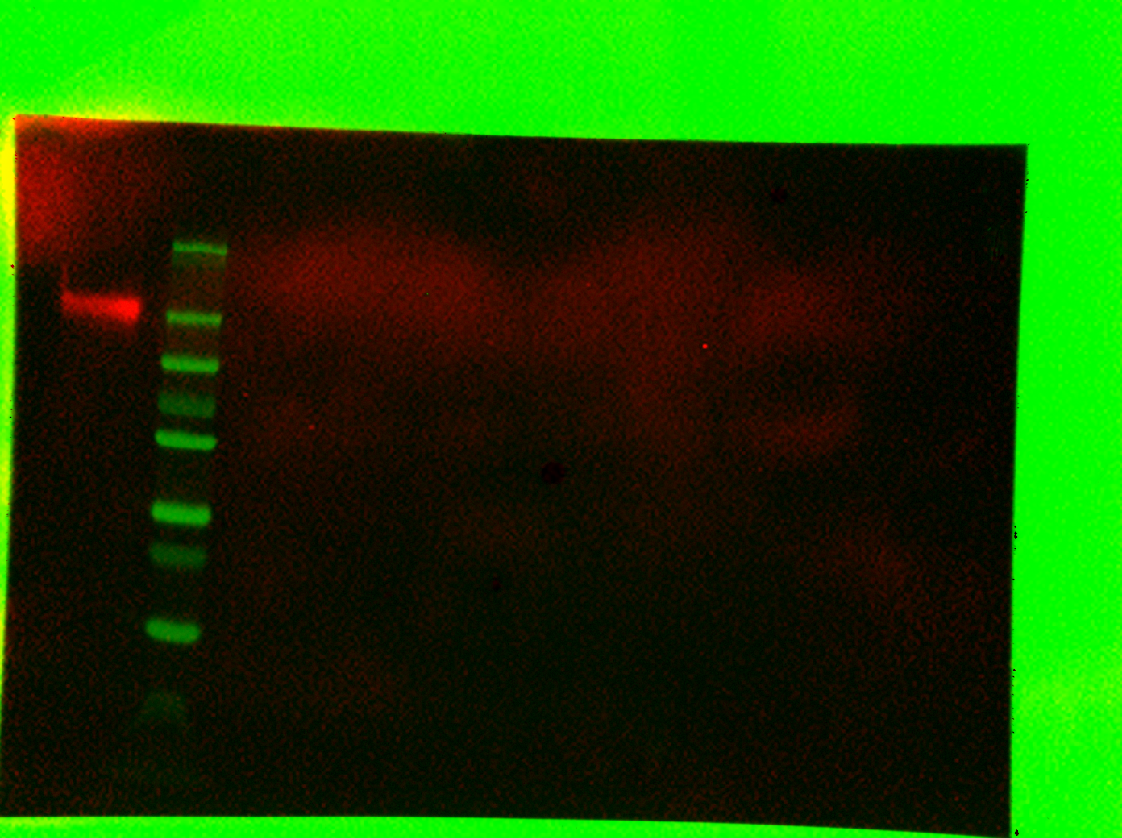

Supplement: Figure 2—figure supplement 1—source data 1. [file elife-82543-fig2-figsupp1-data1.zip › Fig 2 - figure supplement 1 - source file/Fig2 -figure supplement 1D - source file_CD31.tif]

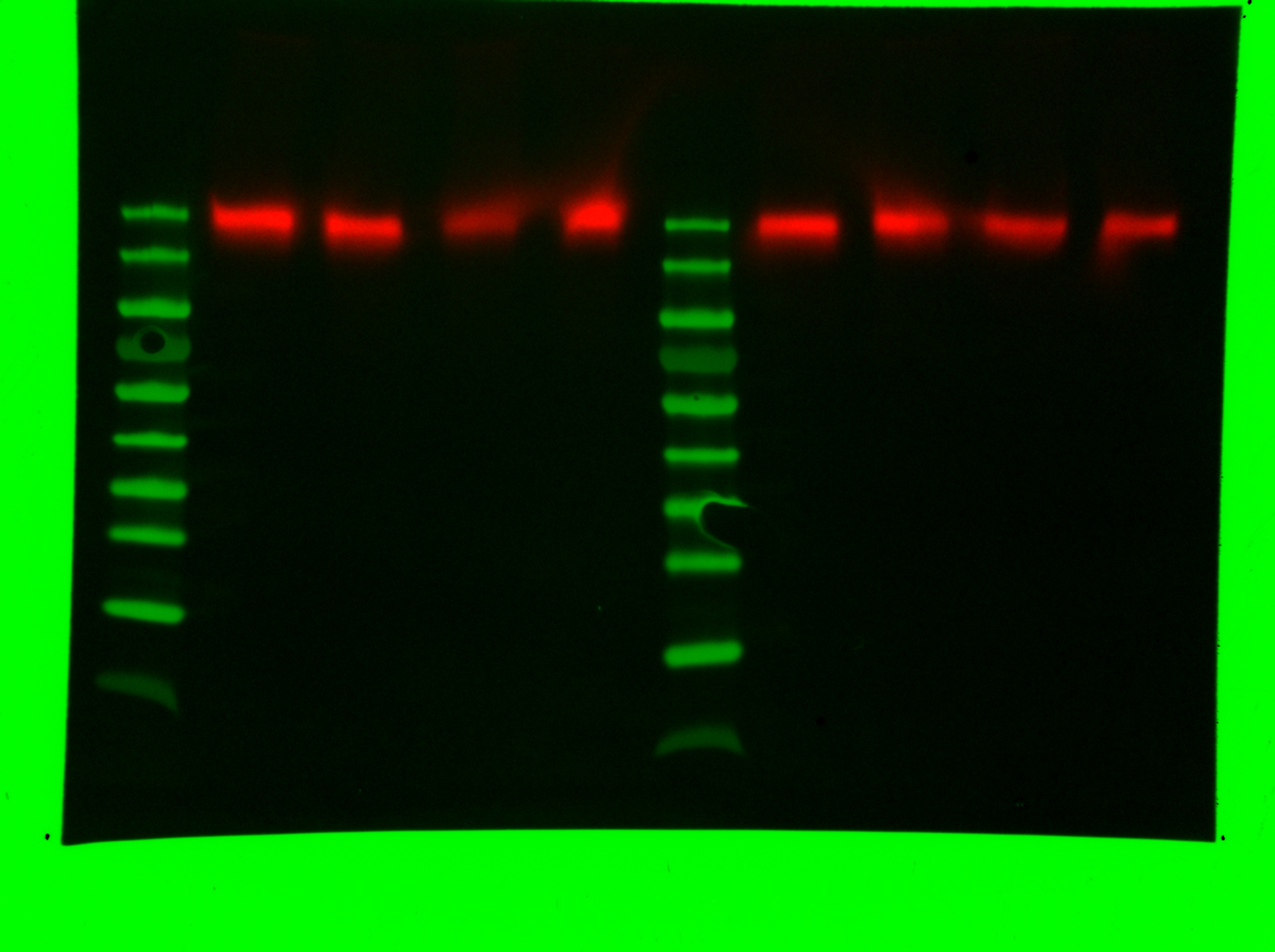

Supplement: Figure 6—source data 1. [file elife-82543-fig6-data1.zip › Fig 6A - source file/Fig 6A - source file_EGFR.tif]

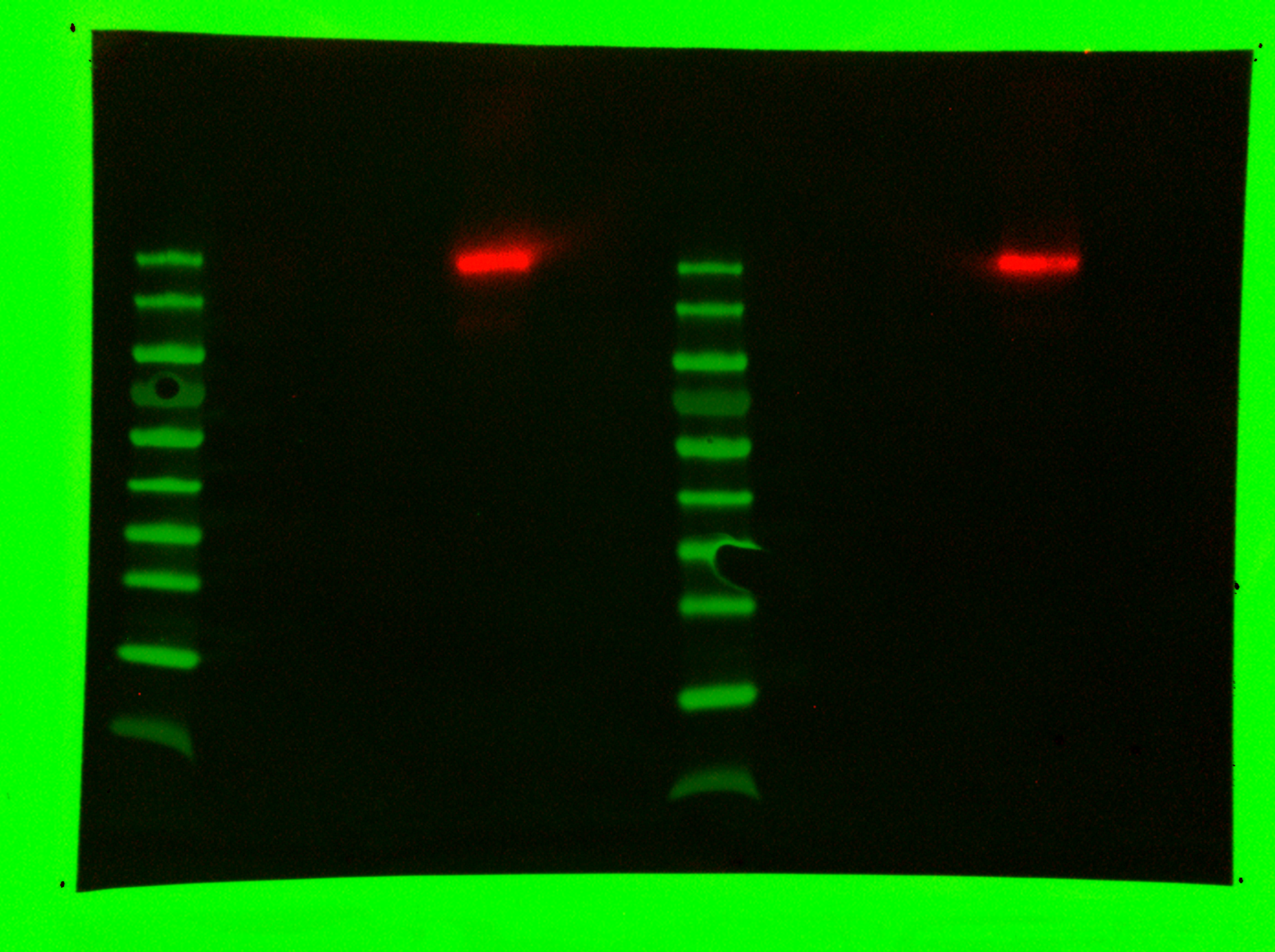

Supplement: Figure 6—source data 1. [file elife-82543-fig6-data1.zip › Fig 6A - source file/Fig 6A - source file_pEGFR.tif]
