# Supplementary material for: ErbB signaling is a potential therapeutic target for vascular lesions with fibrous component
Source: eLife. 2023 May 18;12:e82543. doi: 10.7554/eLife.82543 (PMC10260011; doi:10.7554/eLife.82543)
Supplement: Figure 6—source data 1. [file elife-82543-fig6-data1.zip › Fig 6A - source file/Fig 6 - source file.pdf]

Full unedited gels for Figure 6A

pEGFR

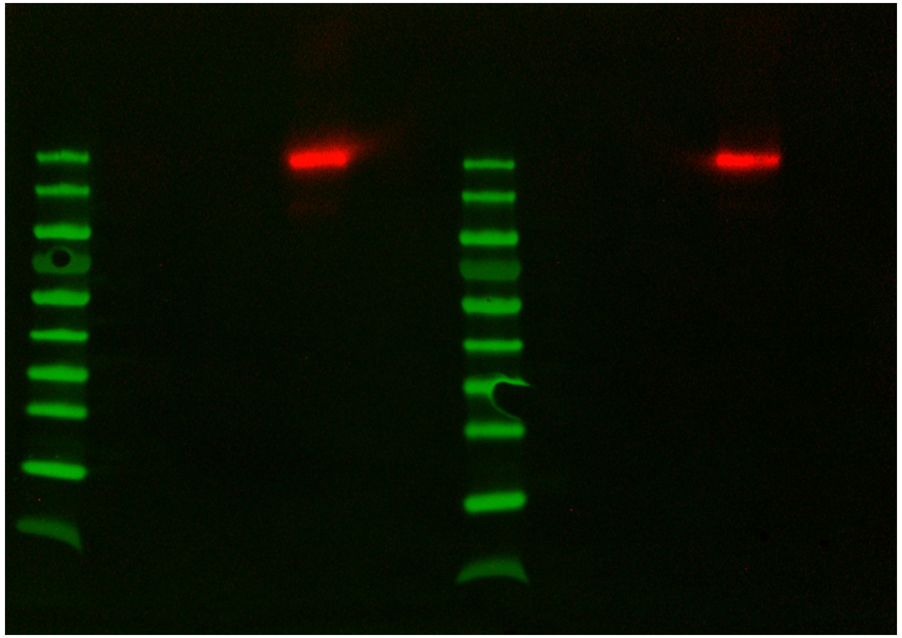

These lanes were included in the Figure

Total EGFR

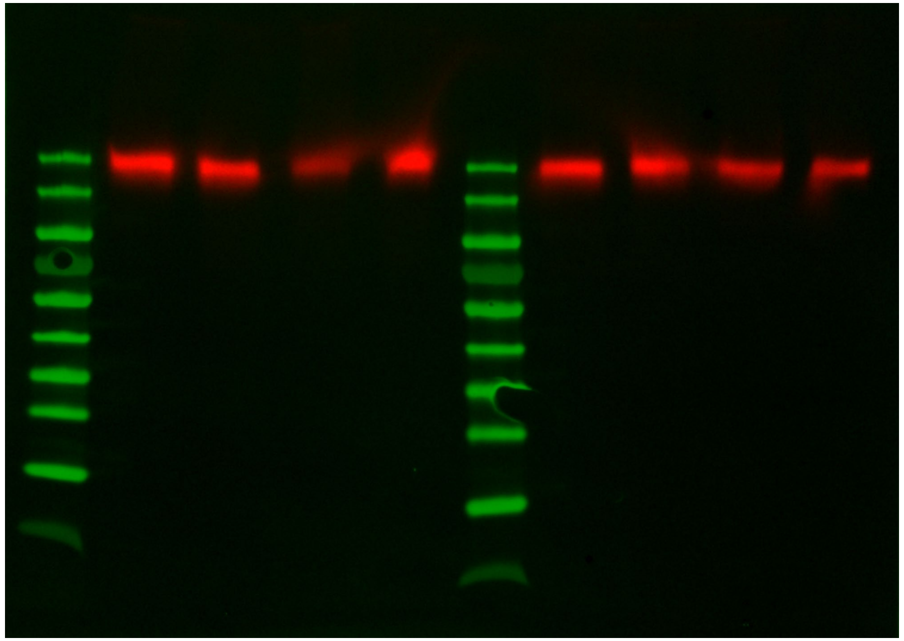

These lanes were included in the Figure
