# Supplementary material for: ErbB signaling is a potential therapeutic target for vascular lesions with fibrous component
Source: eLife. 2023 May 18;12:e82543. doi: 10.7554/eLife.82543 (PMC10260011; doi:10.7554/eLife.82543)
Supplement: Supplementary file 2. [file elife-82543-supp2.docx]

**gBlocks Gene Fragments - used as positive controls in ddPCR**

PIK3CA c.1624G>A (p.E542K) WT

CAGAGTAACAGACTAGCTAGAGACAATGAATTAAGGGAAAATGACAAAGAACAGCTCAAAGCAATTTCTACACGAGATCCTCTCTCTGAAATCACTGAGCAGGAGAAAGATTTTCTATGGAGTCACAGGTAAGTGCTAAAATGGAGATTCTCTGTTTCTTTTTCTTTATTACAGAAAAAATAACTGAATTTGGCTGATCTCAGCATGTTTTTACCATACCTATTGG

PIK3CA c.1624G>A (p.E542K) Mut

CAGAGTAACAGACTAGCTAGAGACAATGAATTAAGGGAAAATGACAAAGAACAGCTCAAAGCAATTTCTACACGAGATCCTCTCTCTAAAATCACTGAGCAGGAGAAAGATTTTCTATGGAGTCACAGGTAAGTGCTAAAATGGAGATTCTCTGTTTCTTTTTCTTTATTACAGAAAAAATAACTGAATTTGGCTGATCTCAGCATGTTTTTACCATACCTATTGG

PIK3CA c.1633G>A (p.E545K) WT

CAGAGTAACAGACTAGCTAGAGACAATGAATTAAGGGAAAATGACAAAGAACAGCTCAAAGCAATTTCTACACGAGATCCTCTCTCTGAAATCACTGAGCAGGAGAAAGATTTTCTATGGAGTCACAGGTAAGTGCTAAAATGGAGATTCTCTGTTTCTTTTTCTTTATTACAGAAAAAATAACTGAATTTGGCTGATCTCAGCATGTTTTTACCATACCTATTGG

PIK3CA c.1633G>A (p.E545K) Mut

CAGAGTAACAGACTAGCTAGAGACAATGAATTAAGGGAAAATGACAAAGAACAGCTCAAAGCAATTTCTACACGAGATCCTCTCTCTGAAATCACTAAGCAGGAGAAAGATTTTCTATGGAGTCACAGGTAAGTGCTAAAATGGAGATTCTCTGTTTCTTTTTCTTTATTACAGAAAAAATAACTGAATTTGGCTGATCTCAGCATGTTTTTACCATACCTATTGG

PIK3CA c.3140A>G (p.H1047R) WT

GATGACATTGCATACATTCGAAAGACCCTAGCCTTAGATAAAACTGAGCAAGAGGCTTTGGAGTATTTCATGAAACAAATGAATGATGCACATCATGGTGGCTGGACAACAAAAATGGATTGGATCTTCCACACAATTAAACAGCATGCATTGAACTGAAAAGATAACTGAGAAAATGAAAGCTCACTCTGGATTCCACACTGCACTGTTAATAACTCTCAGCAGGCAAAGACCGATTGCATAGGAATTGCACAATCCATGAACAGCATTAG

PIK3CA c.3140A>G (p.H1047R) Mut

GATGACATTGCATACATTCGAAAGACCCTAGCCTTAGATAAAACTGAGCAAGAGGCTTTGGAGTATTTCATGAAACAAATGAATGATGCACGTCATGGTGGCTGGACAACAAAAATGGATTGGATCTTCCACACAATTAAACAGCATGCATTGAACTGAAAAGATAACTGAGAAAATGAAAGCTCACTCTGGATTCCACACTGCACTGTTAATAACTCTCAGCAGGCAAAGACCGATTGCATAGGAATTGCACAATCCATGAACAGCATTAG

PIK3CA c.3140A>T (p.H1047L) WT

GATGACATTGCATACATTCGAAAGACCCTAGCCTTAGATAAAACTGAGCAAGAGGCTTTGGAGTATTTCATGAAACAAATGAATGATGCACATCATGGTGGCTGGACAACAAAAATGGATTGGATCTTCCACACAATTAAACAGCATGCATTGAACTGAAAAGATAACTGAGAAAATGAAAGCTCACTCTGGATTCCACACTGCACTGTTAATAACTCTCAGCAGGCAAAGACCGATTGCATAGGAATTGCACAATCCATGAACAGCATTAG

PIK3CA c.3140A>T (p.H1047L) Mut

GATGACATTGCATACATTCGAAAGACCCTAGCCTTAGATAAAACTGAGCAAGAGGCTTTGGAGTATTTCATGAAACAAATGAATGATGCACTTCATGGTGGCTGGACAACAAAAATGGATTGGATCTTCCACACAATTAAACAGCATGCATTGAACTGAAAAGATAACTGAGAAAATGAAAGCTCACTCTGGATTCCACACTGCACTGTTAATAACTCTCAGCAGGCAAAGACCGATTGCATAGGAATTGCACAATCCATGAACAGCATTAG

TEK c.2740C>T (p.L914F) WT

TGTTTTTTAGACAAAATGGAGTTTATAGGCAATTTCCACAGCACATCTCTTAAATGTCATAGCTGTTCAGGGCCACTGATGAGTCGATGCTCTCTTCCTTCCCTCCAGGCTACTTGTACCTGGCCATTGAGTACGCGCCCCATGGAAACCTTCTGGACTTCCTTCGCAAGAGCCGTGTGCTGGAGACGGACCCAGCATTTGCCATTGCCAATAGCACCGCGTCCACACTGTCCTCCCAGCAGCTCCTTCACTTCGCTGCCGACGTGGCCCGGGGCATGGACTACTTGAGCCAAAAACAGG

TEK c.2740C>T (p.L914F) Mut

TGTTTTTTAGACAAAATGGAGTTTATAGGCAATTTCCACAGCACATCTCTTAAATGTCATAGCTGTTCAGGGCCACTGATGAGTCGATGCTCTCTTCCTTCCCTCCAGGCTACTTGTACCTGGCCATTGAGTACGCGCCCCATGGAAACCTTCTGGACTTCTTTCGCAAGAGCCGTGTGCTGGAGACGGACCCAGCATTTGCCATTGCCAATAGCACCGCGTCCACACTGTCCTCCCAGCAGCTCCTTCACTTCGCTGCCGACGTGGCCCGGGGCATGGACTACTTGAGCCAAAAACAGG
