# Supplementary material for: ErbB signaling is a potential therapeutic target for vascular lesions with fibrous component
Source: eLife. 2023 May 18;12:e82543. doi: 10.7554/eLife.82543 (PMC10260011; doi:10.7554/eLife.82543)
Supplement: MDAR checklist [file elife-82543-mdarchecklist1.docx]

**Materials Design Analysis Reporting (MDAR)**

**Checklist for Authors**

The [MDAR framework](https://osf.io/xfpn4/) establishes a minimum set of requirements in transparent reporting mainly applicable to studies in the life sciences.

*eLife* asks authors to **provide detailed information within their article** to facilitate the interpretation and replication of their work. Authors can also upload supporting materials to comply with relevant reporting guidelines for health-related research (see [EQUATOR Network](http://www.equator-network.org/%20)), life science research (see the [BioSharing Information Resource](http://biosharing.org/)), or animal research (see the [ARRIVE Guidelines](http://www.plosbiology.org/article/info:doi/10.1371/journal.pbio.1000412) and the [STRANGE Framework](https://doi.org/10.1038/d41586-020-01751-5); for details, see *eLife*’s [Journal Policies](https://reviewer.elifesciences.org/author-guide/journal-policies)). Where applicable, authors should refer to any relevant reporting standards materials in this form.

For all that apply, please note **where in the article** the information is provided. Please note that we also collect information about data availability and ethics in the submission form.

**Materials:**

| **Newly created materials** | **Indicate where provided: section/figure legend** | **N/A** |
| --- | --- | --- |
| The manuscript includes a dedicated "materials availability statement" providing transparent disclosure about availability of newly created materials including details on how materials can be accessed and describing any restrictions on access. | This information can be found in the “RNA-sequencing and gene ontology analysis” section of Materials and Methods and in the Availability of Data and Material section. RNA-seq data has been submitted to NCBI Gene Expression Omnibus under accession numbers GSE130807 and GSE196311. Private links for reviewers: Go to https://www.ncbi.nlm.nih.gov/geo/query/acc.cgi?acc= GSE130807 or https://www.ncbi.nlm.nih.gov/geo/query/acc.cgi?acc= GSE196311. Enter token wbivkayaxhojdqp or mbehiikgvtmfryh into the box, respectively. |  |
|  |  |  |
| **Antibodies** | **Indicate where provided: section/figure legend** | **N/A** |
| For commercial reagents, provide supplier name, catalogue number and [RRID](https://scicrunch.org/resources), if available. | This information can be found in the Materials and Methods under specific sections related to each experiment. |  |
|  |  |  |
| **DNA and RNA sequences** | **Indicate where provided: section/figure legend** | **N/A** |
| Short novel DNA or RNA including primers, probes: Sequences should be included or deposited in a public repository. | Information about primers used in RT-qPCR experiments can be found in the “Table 4. Taqman assays used in RT-qPCR”. Information about primers and probes used in mutational analysis can be found in the “Mutational analysis” section of Materials and Methods. Information about sequences of synthetic gBlock gene fragments used as positive controls in the mutation analysis can be found in Supplementary Material 2. |  |
|  |  |  |
| **Cell materials** | **Indicate where provided: section/figure legend** | **N/A** |
| Cell lines: Provide species information, strain. Provide accession number in repository OR supplier name, catalog number, clone number, OR RRID. |  | N/A |
| Primary cultures: Provide species, strain, sex of origin, genetic modification status. | This information can be found in the “Cell culture” section of Materials and Methods. In this study we used human primary CD31+ endothelial cells and CD31-/Vimentin+ stromal cells isolated from patients’ vascular lesions. Background information of the patients can be found in the Tables 1 and 2. Human endothelial cells were extracted from umbilical cords in our own laboratory and cells from 3 donors were used in the experiments. In addition, commercially available human control cells (HsaVEC and HPF-c) each from 3 donors were used in the experiments. |  |
|  |  |  |
| **Experimental animals** | **Indicate where provided: section/figure legend** | **N/A** |
| Laboratory animals or Model organisms: Provide species, strain, sex, age, genetic modification status. Provide accession number in repository OR supplier name, catalog number, clone number, OR RRID. | This information can be found in the “A modified xenograft model for vascular lesion” section of Materials and Methods. 6-weeks old female Hsd:Athymic Nude-Foxn1nu mice from Envigo (Indiana, USA) were used in the experiments. |  |
| Animal observed in or captured from the field: Provide species, sex, and age where possible. |  | N/A |
|  |  |  |
| **Plants and microbes** | **Indicate where provided: section/figure legend** | **N/A** |
| Plants: provide species and strain, ecotype and cultivar where relevant, unique accession number if available, and source (including location for collected wild specimens). |  | N/A |
| Microbes: provide species and strain, unique accession number if available, and source. |  | N/A |
|  |  |  |
| **Human research participants** | **Indicate where provided: section/figure legend) or state if these demographics were not collected** | **N/A** |
| If collected and within the bounds of privacy constraints report on age, sex, gender and ethnicity for all study participants. | This information can be found in the “Patient cohort” section of Materials and Methods and in more details in the Tables 1 and 2. |  |

**Design:**

| **Study protocol** | **Indicate where provided: section/figure legend** | **N/A** |
| --- | --- | --- |
| If the study protocol has been pre-registered, provide DOI. For clinical trials, provide the trial registration number OR cite DOI. |  | N/A |
|  |  |  |
| **Laboratory protocol** | **Indicate where provided: section/figure legend** | **N/A** |
| Provide DOI OR other citation details if detailed step-by-step protocols are available. |  | N/A |
|  |  |  |
| **Experimental study design (statistics details) *** | | |
| **For in vivo studies: State whether and how the following have been done** | **Indicate where provided: section/figure legend. If it could have been done, but was not, write “not done”** | **N/A** |
| Sample size determination | No power calculations were made as the study was exploratory in nature with very limited previous data to support the calculations. Sample sizes were determined in part by feasibility. |  |
| Randomisation | This information can be found in the “A modified xenograft model for vascular lesion” section of Materials and Methods. For comparison lesion growth with or without oncogenic PIK3CA variant, each mouse had two matrigel plugs: one with PIK3CAH1047R ECs and one with PIK3CAwt ECs. Prior injections, mice were randomised to groups receiving either ECs+FBs or ECs, or to be treated with or without afatinib . |  |
| Blinding | This information can be found in the “Fibrin bead assay” and “A modified xenograft model for vascular lesion” sections of Materials and Methods. Histological sections of matrigel plugs and images of fibrin beads were analyzed in a blinded manner either by 2 independent observers (fibrin bead assay, CD31 IHC for matrigel plugs) or by one observer (scoring on H&E stained sections of matrigel plugs). |  |
| Inclusion/exclusion criteria | This information can be found in the “A modified xenograft model for vascular lesion” section of Materials and Methods. Only mice with lesion growth at day 9 were included in the afatinib study. |  |
|  |  |  |
| **Sample definition and in-laboratory replication** | **Indicate where provided: section/figure legend** | **N/A** |
| State number of times the experiment was replicated in the laboratory. | This information can be found in the Materials and Methods under specific sections related to each experiment as well as in the figure legends describing each individual experiment. |  |
| Define whether data describe technical or biological replicates. | This information can be found in the Materials and Methods under specific sections related to each experiment. |  |
|  |  |  |
| **Ethics** | **Indicate where provided: section/submission form** | **N/A** |
| Studies involving human participants: State details of authority granting ethics approval (IRB or equivalent committee(s), provide reference number for approval. | This information can be found in the “Patient cohort” section of Materials and Methods. Regarding vascular anomalies, patient sample collection was approved by the Ethical Committee of the Helsinki University hospital, Helsinki, Finland (Decision No 127/13/03/02/2010 and No 1394/2020). The control sample collection was approved by the Research Ethics Committee of the Northern Savo Hospital District, Kuopio, Finland (Decision No 139/2015). |  |
| Studies involving experimental animals: State details of authority granting ethics approval (IRB or equivalent committee(s), provide reference number for approval. | This information can be found in the “A modified xenograft model for vascular lesion” section of Materials and Methods. Animal experiments were approved by National Experimental Animal Board of Finland (Decision No Esavi-2019-004672) and carried out in accordance with guidelines of the Finnish Act on Animal Experimentation. |  |
| Studies involving specimen and field samples: State if relevant permits obtained, provide details of authority approving study; if none were required, explain why. | This information can be found in the “Cell culture” section of Materials and Methods. Umbilical cord collection for HUVEC isolation was performed with approval from the Research Ethics Committee of the Northern Savo Hospital District, Kuopio, Finland (Decision No 341/2015). |  |
|  |  |  |
| **Dual Use Research of Concern (DURC)** | **Indicate where provided: section/submission form** | **N/A** |
| If study is subject to dual use research of concern regulations, state the authority granting approval and reference number for the regulatory approval. |  | N/A |

**Analysis:**

| **Attrition** | **Indicate where provided: section/figure legend** | **N/A** |
| --- | --- | --- |
| Describe whether exclusion criteria were pre-established. Report if sample or data points were omitted from analysis. If yes, report if this was due to attrition or intentional exclusion and provide justification. | All patients having AST or VM were included in the study.    In relation to animal experiments, this information can be found in the “A modified xenograft model for vascular lesion” section of Materials and Methods. Exclusion criteria from the analysis were:  i) unsuccessful plug formation; and ii) different anatomical location of the plug. |  |
|  |  |  |
| **Statistics** | **Indicate where provided: section/figure legend** | **N/A** |
| Describe statistical tests used and justify choice of tests. | This information can be found in the “Statistical analysis” section of Materials and Methods. |  |
|  |  |  |
| **Data availability** | **Indicate where provided: section/submission form** | **N/A** |
| For newly created and reused datasets, the manuscript includes a data availability statement that provides details for access (or notes restrictions on access). | The data availability statement is included in the manuscript on page 24, after the Discussion. |  |
| When newly created datasets are publicly available, provide accession number in repository OR DOI and licensing details where available. | This information can be found in the “RNA-sequencing and gene ontology analysis” section of Materials and Methods and in the Availability of Data and Material section. RNA-seq data has been submitted to NCBI Gene Expression Omnibus under accession numbers GSE130807 and GSE196311. Private links for reviewers: Go to https://www.ncbi.nlm.nih.gov/geo/query/acc.cgi?acc= GSE130807 or https://www.ncbi.nlm.nih.gov/geo/query/acc.cgi?acc= GSE196311. Enter token wbivkayaxhojdqp or mbehiikgvtmfryh into the box, respectively. |  |
| If reused data is publicly available provide accession number in repository OR DOI, OR URL, OR citation. | Information about reused data can be found in the “Oncogenic PIK3CA p.H1047R induces expression of TGFA and enrichment of hallmark hypoxia” section of Results. RNA-seq data from ECs expressing constitutively active hypoxia inducible factors is available in NCBI Gene Expression Omnibus under accession number GSE98060. Go to https://www.ncbi.nlm.nih.gov/geo/query/acc.cgi?acc= GSE98060. The data is publicly available, and no token is needed to open it. |  |
|  |  |  |
| **Code availability** | **Indicate where provided: section/figure legend** | **N/A** |
| For any computer code/software/mathematical algorithms essential for replicating the main findings of the study, whether newly generated or re-used, the manuscript includes a data availability statement that provides details for access or notes restrictions. |  | N/A |
| Where newly generated code is publicly available, provide accession number in repository, OR DOI OR URL and licensing details where available. State any restrictions on code availability or accessibility. |  | N/A |
| If reused code is publicly available provide accession number in repository OR DOI OR URL, OR citation. |  | N/A |

**Reporting:**

The MDAR framework recommends adoption of discipline-specific guidelines, established and endorsed through community initiatives.

| **Adherence to community standards** | **Indicate where provided: section/figure legend** | **N/A** |
| --- | --- | --- |
| State if relevant guidelines (e.g., ICMJE, MIBBI, ARRIVE, STRANGE) have been followed, and whether a checklist (e.g., CONSORT, PRISMA, ARRIVE) is provided with the manuscript. | Relevant guidelines have been followed (ARRIVE). |  |

* We provide the following guidance regarding transparent reporting and statistics; we also refer authors to [Ten common statistical mistakes to watch out for when writing or reviewing a manuscript](https://doi.org/10.7554/eLife.48175).

**Sample-size estimation**

- You should state whether an appropriate sample size was computed when the study was being designed
- You should state the statistical method of sample size computation and any required assumptions
- If no explicit power analysis was used, you should describe how you decided what sample (replicate) size (number) to use

**Replicates**

- You should report how often each experiment was performed
- You should include a definition of biological versus technical replication
- The data obtained should be provided and sufficient information should be provided to indicate the number of independent biological and/or technical replicates
- If you encountered any outliers, you should describe how these were handled
- Criteria for exclusion/inclusion of data should be clearly stated
- High-throughput sequence data should be uploaded before submission, with a private link for reviewers provided (these are available from both GEO and ArrayExpress)

**Statistical reporting**

- Statistical analysis methods should be described and justified
- Raw data should be presented in figures whenever informative to do so (typically when N per group is less than 10)
- For each experiment, you should identify the statistical tests used, exact values of N, definitions of center, methods of multiple test correction, and dispersion and precision measures (e.g., mean, median, SD, SEM, confidence intervals; and, for the major substantive results, a measure of effect size (e.g., Pearson's r, Cohen's d)
- Report exact p-values wherever possible alongside the summary statistics and 95% confidence intervals. These should be reported for all key questions and not only when the p-value is less than 0.05.

**Group allocation**

- Indicate how samples were allocated into experimental groups (in the case of clinical studies, please specify allocation to treatment method); if randomization was used, please also state if restricted randomization was applied
- Indicate if masking was used during group allocation, data collection and/or data analysis
